# Differential expression and analysis of extrachromosomal circular DNAs as serum biomarkers in pulmonary arterial hypertension

**DOI:** 10.1186/s12931-024-02808-z

**Published:** 2024-04-25

**Authors:** Chun Zhang, Qiang Du, Xiao Zhou, Tianyu Qu, Yingying Liu, Kai Ma, Ziling Shen, Qun Wang, Zaikui Zhang, Ruifeng Zhang

**Affiliations:** 1 Department of Respiratory Medicine, Zhongda Hospital of Southeast University, Dingjiaqiao 87, Nanjing City, Jiangsu Province 210000 People’s Republic of China; 2 Center of Pathology and Clinical Laboratory, Sir Run Run Hospital, Nanjing Medical University, Nanjing, 210000 People’s Republic of China

**Keywords:** EccDNA, Pulmonary arterial hypertension, High-throughput sequencing, Differential expression, Biomarker

## Abstract

**Background:**

Extrachromosomal circular DNAs (eccDNAs) have been reported to play a key role in the occurrence and development of various diseases. However, the characterization and role of eccDNAs in pulmonary arterial hypertension (PAH) remain unclear.

**Methods:**

In the discovery cohort, we first explored eccDNA expression profiles by Circle-sequencing analysis. The candidate eccDNAs were validated by routine polymerase chain reaction (PCR), TOPO-TA cloning and Sanger sequencing. In the validation cohort, 30 patients with PAH and 10 healthy controls were recruited for qPCR amplification to detect the candidate eccDNAs. Datas at the baseline were collected, including clinical background, biochemical variables, echocardiography and hemodynamic factors. Receiver operating characteristic curve was used to investigate the diagnostic effect of the eccDNA.

**Results:**

We identified a total of 21,741 eccDNAs in plasma samples of 3 IPAH patients and 3 individuals in good health, and the expression frequency, GC content, length distribution, and genome distribution of the eccDNAs were thoroughly characterized and analyzed. In the validation cohort, 687 eccDNAs were differentially expressed in patients with IPAH compared with healthy controls (screening threshold: |FC|≥2 and *P* < 0.05). Gene Ontology (GO) and the Kyoto Encyclopedia of Genes and Genomes (KEGG) pathway analysis showed that the specific eccDNAs in IPAH were significantly enriched in calcium channel activity, the mitogen-activated protein kinase pathway, and the wnt signaling pathway. Verification queue found that the expression of eccDNA-chr2:131208878–131,424,362 in PAH was considerably higher than that in healthy controls and exhibited a high level of accuracy in predicting PAH with a sensitivity of 86.67% and a specificity of 90%. Furthermore, correlation analysis disclosed a significant association between serum eccDNA-chr2:131208878–131,424,362 and mean pulmonary artery pressure (mPAP) (*r* = 0.396, *P* = 0.03), 6 min walking distance (6MWD) (*r* = -0.399, *P* = 0.029), N-terminal pro-B-type natriuretic peptide (NT-proBNP) (*r* = 0.685, *P* < 0.001) and cardiac index (CI) (*r* = − 0.419, *P* = 0.021).

**Conclusions:**

This is the first study to identify and characterize eccDNAs in patients with PAH. We revealed that serum eccDNA-chr2:131208878–131,424,362 is significantly overexpressed and can be used in the diagnosis of PAH, indicating its potential as a novel non-invasive biomarker.

**Supplementary Information:**

The online version contains supplementary material available at 10.1186/s12931-024-02808-z.

## Introduction

Pulmonary arterial hypertension (PAH) is a fatal pulmonary vascular disease which is marked by elevated pressure and the failure of the right ventricle [1, 2]. Despite the significant progress in enhancing survival through the advancement of novel treatments, PAH remains a medical condition with high mortality rates [2]. The timely identification and predictive assessment of PAH may guide decisions regarding the use of more aggressive therapy; nevertheless, evaluating the prognosis of PAH is challenging and predominantly based on invasive hemodynamics like right heart catheterization [3]. Uric acid [4], troponin T [5], and brain natriuretic peptide [6, 7] have been proposed as prognostic indicators in PAH, but there remains a requirement to discover additional innovative, precise, and non-intrusive biomarkers for the diagnosis of PAH.

In recent years, there has been a growing interest in a particular group of circular DNA molecules called extrachromosomal circular DNAs (eccDNAs), which are commonly present in eukaryotic organisms [8, 9]. EccDNAs are not reliant on the chromosomal genome and play distinct roles in physiological or pathological processes [10, 11]. Studies have indicated that eccDNAs may have a vital part in conditions related to epigenetic modification, such as cancers [12]. Whole-genome sequence analyses have revealed that eccDNAs exhibit a wide range of sizes, varying from less than 100 base pairs to megabases (Mb), and that eccDNAs can carry complete or partial genes and intergenic sequences [13–15]. EccDNAs with a longer size contain intact genes, particularly oncogenic driver genes commonly found in tumors, and uncontrolled expression of these genes ultimately results in the malignant proliferation of tumors [16, 17]. However, there is limited knowledge regarding the roles and mechanisms of the wider range of smaller eccDNAs, since they lack the capacity to include coding sequences. The distribution of sizes for eccDNAs varies between maternal and fetal plasma, with fetal eccDNAs being shorter than maternal eccDNAs [18]. Another study found that the eccDNAs detected in lung cancer specimens were of greater length compared to those in corresponding healthy tissue, and the size of eccDNAs circulating in the plasma diminished following surgical removal of lung cancer [19]. Therefore, eccDNA hold significance not only in terms of functionality but also as a potential biomarker for evaluating disease risk, detecting early signs, and predicting outcomes. Nevertheless, studies on the differential expression and function of eccDNAs in patients with PAH have been lacking up to now.

For this investigation, we employed a high-throughput technique called Circle-Seq to collect and identify eccDNAs from the serum of patients with IPAH and healthy people. We identified a novel eecDNA, eccDNA-chr2:131208878–131,424,362, which showed a substantial increase when compared to healthy individuals. Subsequently, we determined the clinical value of this novel eccDNA.

## Materials and methods

### Clinical specimens

Three patients with incidental IPAH and three healthy participants were recruited as a discovery set. Validation was performed with 30 patients with PAH and 10 healthy controls. The diagnosis of PAH was confirmed based on the guidelines provided by the European Society of Cardiology and the European Respiratory Society [20]. Patients with pulmonary hypertension due to other causes were excluded. Supplementary Table 1 contained the clinical details of all participants.

### Clinical parameter collection

Datas regarding the patients’ age, sex, body mass index (BMI), New York Heart Association (NYHA) functional class, and 6-min walk distance (6MWD) were collected. 6MWD was measured following the established procedure [21]. Biochemical markers, including serum NT-proBNP, uric acid (UA), and troponin I (TnI) were measured using relevant kits (Roche Diagnostics, Germany). Left ventricular ejection fraction (LVEF), left atrial dimension, right atrial dimension, left ventricular dimension, right ventricular dimension, pulmonary artery internal dimension, mitral orifice flow velocity, aortic orifice blood flow velocity and pulmonary artery blood flow were obtained from echocardiographic examinations (EPIQ 7 C; Philips Corporation, USA). Measurements in two dimensions and Doppler were acquired and assessed following the protocols set by the American Society of Echocardiography [22]. Cardiac catheterization was used to assess pulmonary capillary wedge pressure (PCWP), mPAP, pulmonary vascular resistance (PVR), CI and cardiac output (CO).

### Circle-sequencing analysis

The procedures for Circle-Seq were in accordance with previously reported methods [23]. Linear DNA was eliminated by treating the DNA with exonuclease V (New England Biolabs) at a temperature of 37℃ for a duration of 5 min. The circular structure of eccDNA was opened by transposable enzymesand the ends of the DNA fragments were attached to the joints. The Klenow enzyme was employed for repairing these gaps and ends. NEBNext® Ultra™ DNA Library Prep Kit (New England Biolabs) was used to create Illumina sequencing libraries, following the instructions provided by the manufacturer. The quality of the library was assessed by employing the Agilent 2100 Bioanalyzer (Agilent Technologies, Inc., USA). The sequencing was performed using Illumina NovaSeq in paired end mode with 150 bp.

### Validation of eccDNAs in the discovery cohort through PCR and Sanger sequencing

Validation experiments were conducted on two eccDNAs that showed significant differential expression in distinct genomic regions and separate chromosomes. The PCR was used to evaluate the expression of eccDNAs, employing Accurate Taq Master Mix (dye plus). The primers for the eccDNAs were designed using the “out-facing” strategy and are listed in Supplementary Table 2. The PCR samples were placed on agarose gels with a concentration of 1.5%. All the amplified products appeared at the right places after separation by agarose gel electrophoresis and corresponded with the expected sizes of the candidates (Supplementary Fig. S1A–B). After purifying the PCR products that had specific positive bands, they were amplified using TOPO-TA cloning and then sent for Sanger sequencing. Confirmation of the circular structure of selected eccDNAs was achieved through agarose (0.6–1.5%) gel electrophoresis for size separation of PCR products and subsequent Sanger sequencing (Supplementary Fig. S2).

### Differential expression verification of candidate eccDNAs in the validation cohort by quantitative PCR

PCR validation of the selected eccDNAs was performed using primers (Supplementary Table S2), considering different conditions such as differential variation and gene location. To begin the quantitative real-time PCR (qPCR), the initial denaturation was performed at a temperature of 95 °C for a duration of 10 min. This was followed by 40 cycles consisting of a denaturation step at 95 °C for 5 s and an annealing step at 60 °C for 30 s. Finally, the PCR was extended at 72 °C for 5 min. The experiments were conducted three times, and the datas of all samples were normalized to a pGEX-5X-2 carrier. Relative expression of eccDNAs was quantified using the comparative threshold cycle value (∆CT) method with the above primers. The relative expression was calculated as fold change = 2−∆(∆Ct) [24].

### Statistical analyses

GraphPad Prism 8.0 and SPSS 22.0 software were used to perform all statistical plots and analyses. The measurement data with normal distribution were expressed as mean ± standard deviation (x ± s), whereas the measurement data with non-normal distribution were expressed as median (M) and interquartile interval M (P25, P75). Counting data were expressed as frequency or percentage (%). Different data indexes of two groups (PAH and N-PAH group) were compared. The two independent samples *t*-test was utilized to compare groups when the measurement data adhered to a normal distribution. Statistically significant differences were observed at a significance level of *P* < 0.05.

## Results

### Features of eccDNAs in IPAH detected by Circle-Seq analysis

**Fig. 1:**
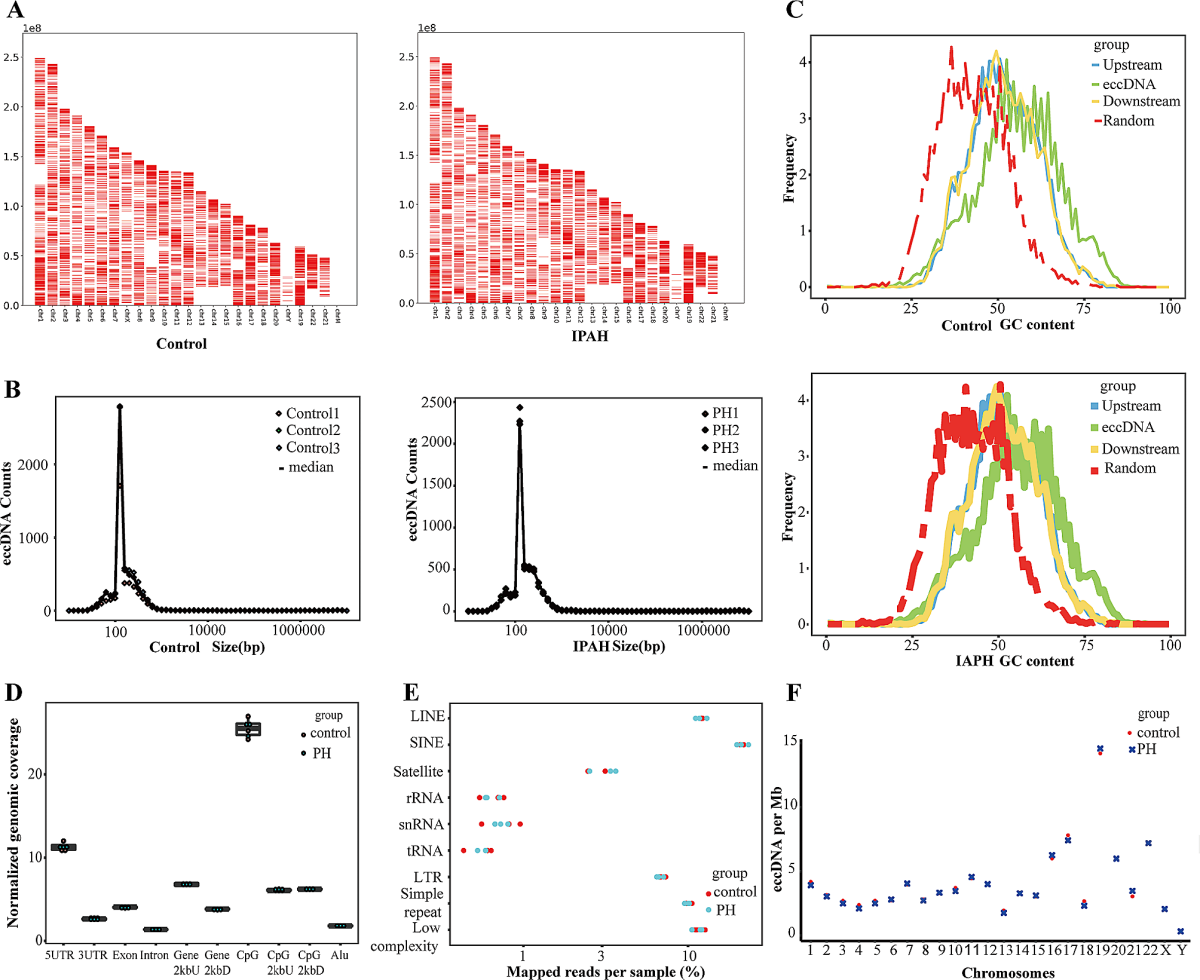
Features of eccDNAs detected in plasma samples from IPAH patients and healthy individuals. **A** Karyotype plots showing the chromosomal distribution of eccDNAs identified in each individual. **B** Size distribution of the identified eccDNAs in the IPAH and Control groups. Individuals are indicated by different color. **C** GC contents of eccDNA locus and regions immediately upstream and downstream of the eccDNA, compared to the genomic average. Blue, 1000 stretches upstream of the eccDNA locus (from eccDNA_start − 1000 to eccDNA_start); green, eccDNA (from eccDNA_start to eccDNA_end); yellow, 1000 stretches downstream of the eccDNA locus (from eccDNA_end to eccDNA_end + 1000); red, 1000 random stretches of the genome of equivalent length as the eccDNA. **D** Genomic distributions of eccDNAs in IPAH and Control groups. CpG2kbD, 2 kb downstream of CpG islands; CpG2kbU, 2 kb upstream of CpG islands; Gene2kbD, 2 kb downstream of genes; Gene2kbU, 2 kb upstream of genes. **E** Repetitive regions from total mapped reads for eccDNAs derived from each sample. Red, Control group; blue, IPAH group. **F** EccDNA frequency relative to chromosome. EccDNA counts per Mb from Control (red circle) and IPAH (green cross) per chromosome

To characterize eccDNA properties in patients with IPAH, a total of 21,741 eccDNAs were detected using Circle-Seq in serum samples from three IPAH patients and three healthy people (see Supplementary Table S3 for quality control data). The expression frequency, length distribution, GC content, and genome distribution of the eccDNAs were characterized and analyzed. The eccDNAs originated from every single chromosome (Fig. 1A). The analysis of size distribution revealed that eccDNAs smaller than 500 bp were the dominant subtypes in the plasma of IPAH patients and healthy participants, with a peak at approximately 140–160 bp in both groups (Fig. 1B). Plasma samples from IPAH patients and healthy individuals showed higher GC contents in eccDNA sequences compared with other regions of the genome (Fig. 1C). This suggests that eccDNAs often possess a high GC content, which aligns with similar findings from other studies [25, 26]. By locating eccDNAs to different genomic elements (Fig. 1D), repeats (Fig. 1E), and different chromosomes (Fig. 1F), we found that eccDNAs are abundant in CpG islands and the 5′ untranslated region (UTR), as well as repeats such as long and short interspersed retrotransposable elements (LINEs and SINEs, respectively). These regions preferentially generate eccDNAs in plasma samples from IPAH patients, rather than regions with a high gene abundance. Concurrently, we found that the gene-rich chromosomes 19 and 17 produced a greater amount of eccDNAs. which suggested that the process of transcription or other characteristics associated with coding genes might contribute to the formation of eccDNAs (Fig. 1F).

### Differential expression profile of eccDNAs in IPAH

**Fig. 2:**
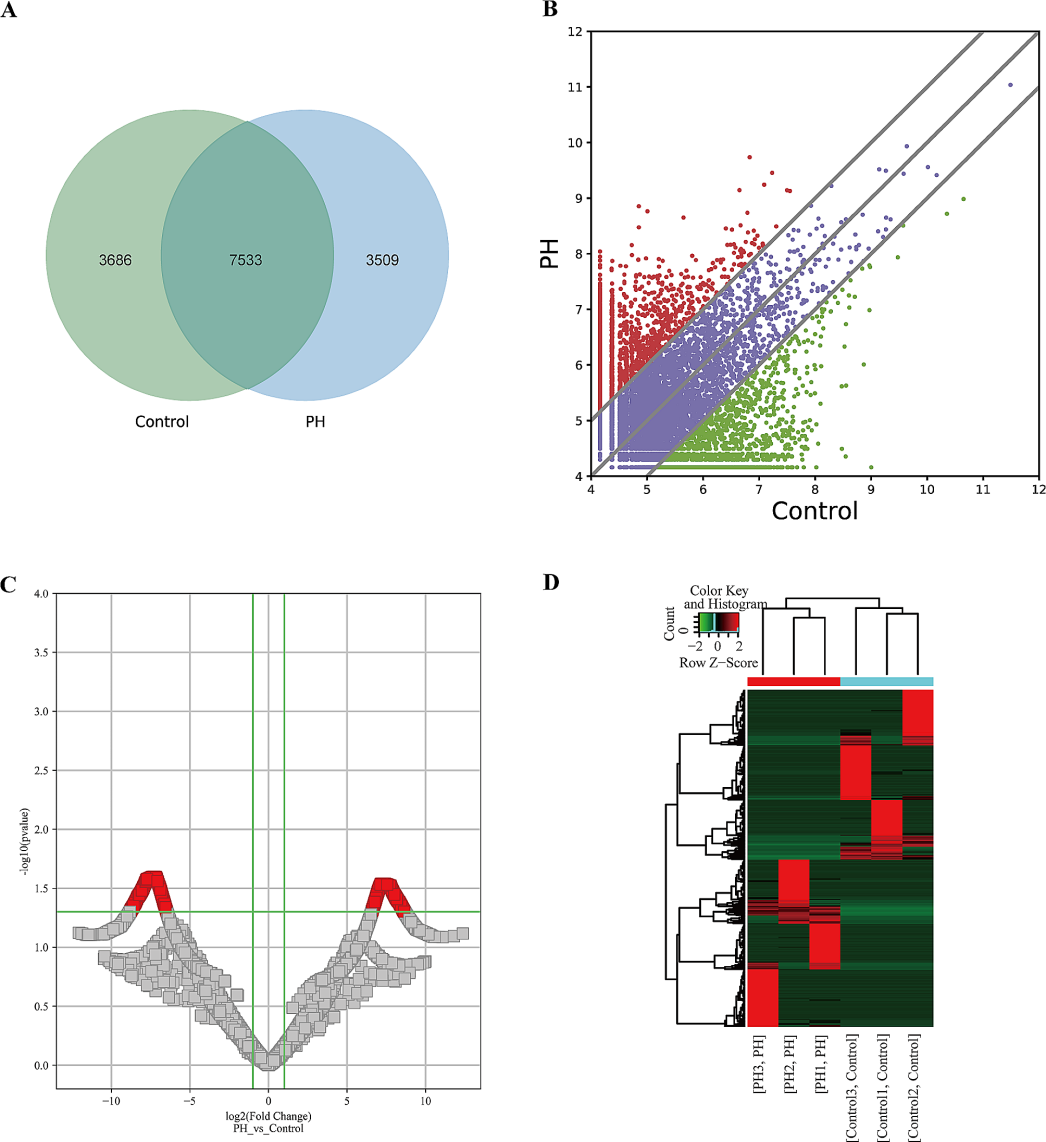
EccDNAs co-expression in IPAH patients and healthy participants and differentially expressed eccDNA genes. **A** The total number of eccDNAs expressed in IPAH patients and healthy participants was 21,741. **B** Dispersion maps of differentially expressed eccDNAs in the IPAH and Control groups. Red dots indicate upregulation, green dots indicate downregulation, and the default multiplicative change threshold was 2.0. **C** Volcano plots were plotted using the fold change and *P*-value when comparing the samples. Red rectangles represent differentially expressed eccDNAs. **D** Heat map and hierarchical clustering of eccDNAs

In order to investigate the possible biological role of eccDNAs in IPAH, the expression profile of the eccDNAs was further analyzed according to the results of Circle-Seq. The total number of eccDNAs expressed in the plasma of IPAH patients and healthy participants was 21,741 (Fig. 2A & Supplementary Table S4). Based on the sequencing results, 687 differentially expressed eccDNAs were screened out in the plasma samples of IPAH patients compared with the healthy participants, with a cutoff standard of |FC (fold change)| ≥ 2 and *P* < 0.05 (Fig. 2B). Among these differentially expressed eccDNAs, 360 were upregulated and 327 were downregulated (Fig. 2C-D & Supplementary Table S5).

### GO and KEGG pathway analyses of genes associated with the differentially expressed eccDNAs

**Fig. 3:**
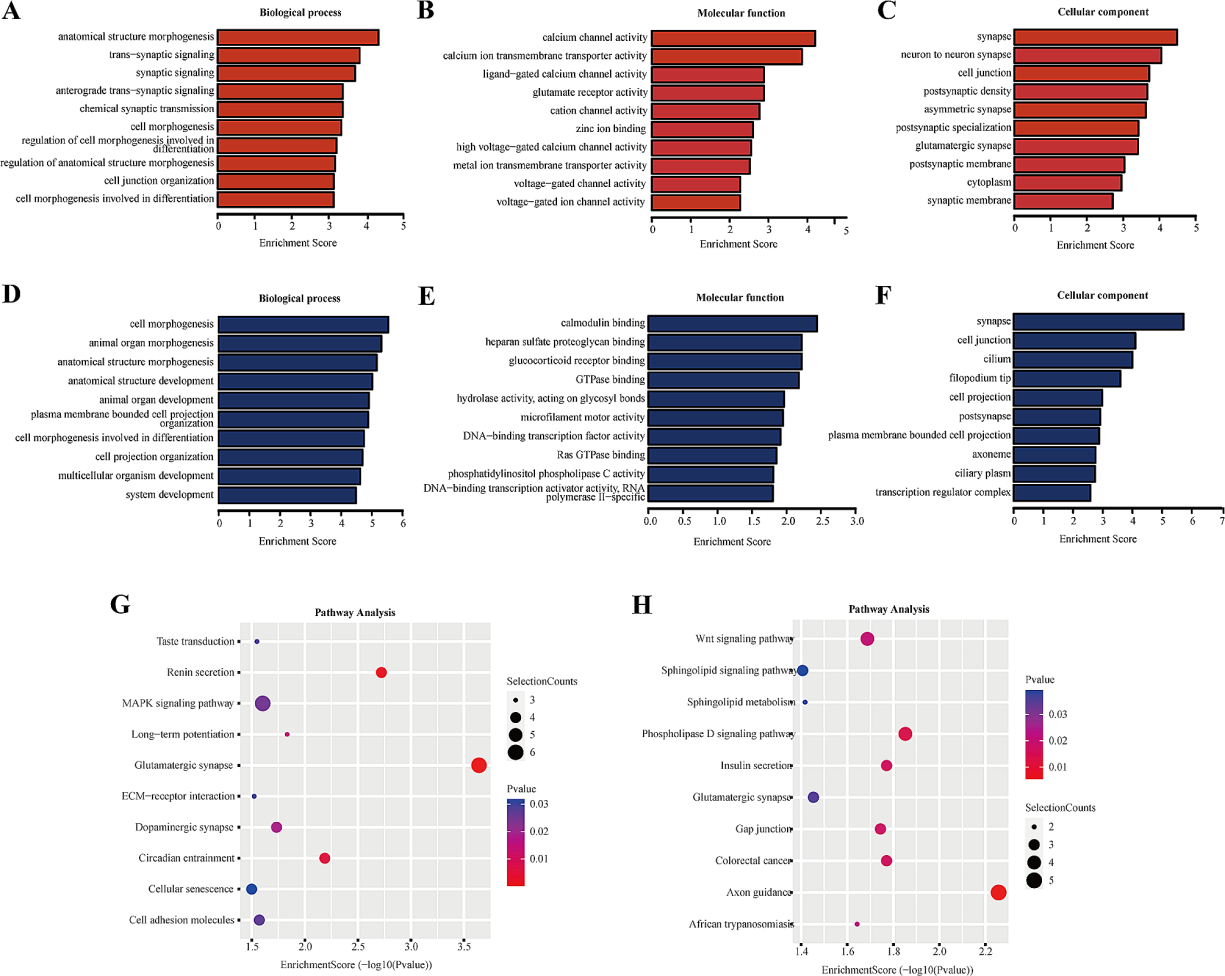
GO and KEGG pathway analyses of the genes associated with the differentially expressed eccDNAs. **A-C** The biological processes, molecular functions, and cellular components associated with the upregulated eccDNAs. **D-F** The biological processes, molecular functions, and cellular components associated with the downregulated eccDNAs. **G** Sixteen upregulated pathways of differentially expressed eccDNA-related mRNA genes revealed in KEGG pathway analysis. **H** Sixteen downregulated pathways of differentially expressed eccDNA-related mRNA genes revealed in KEGG pathway analysis

To examine the functions of the genes associated with the differentially expressed eccDNAs, GO analysis was performed, including identification of the related biological processes, molecular functions, and cellular components (Fig. 3A–F). The dominant biological processes were related to cell morphogenesis and anatomical structure morphogenesis, and the main molecular function and cellular component was calcium channel activity and synapse, respectively. According to the KEGG pathway analysis, the genes associated with the differentially expressed eccDNAs were primarily linked to glutamatergic synapse, the mitogen-activated protein kinase (MAPK) pathway, the phospholipase D signaling pathway, axon guidance, and the wnt signaling pathway (Fig. 3G-H).

### Serum eccDNA-chr2:131208878–131,424,362 was elevated in patients with PAH and could serve as a potential serum biomarker of PAH

**Fig. 4:**
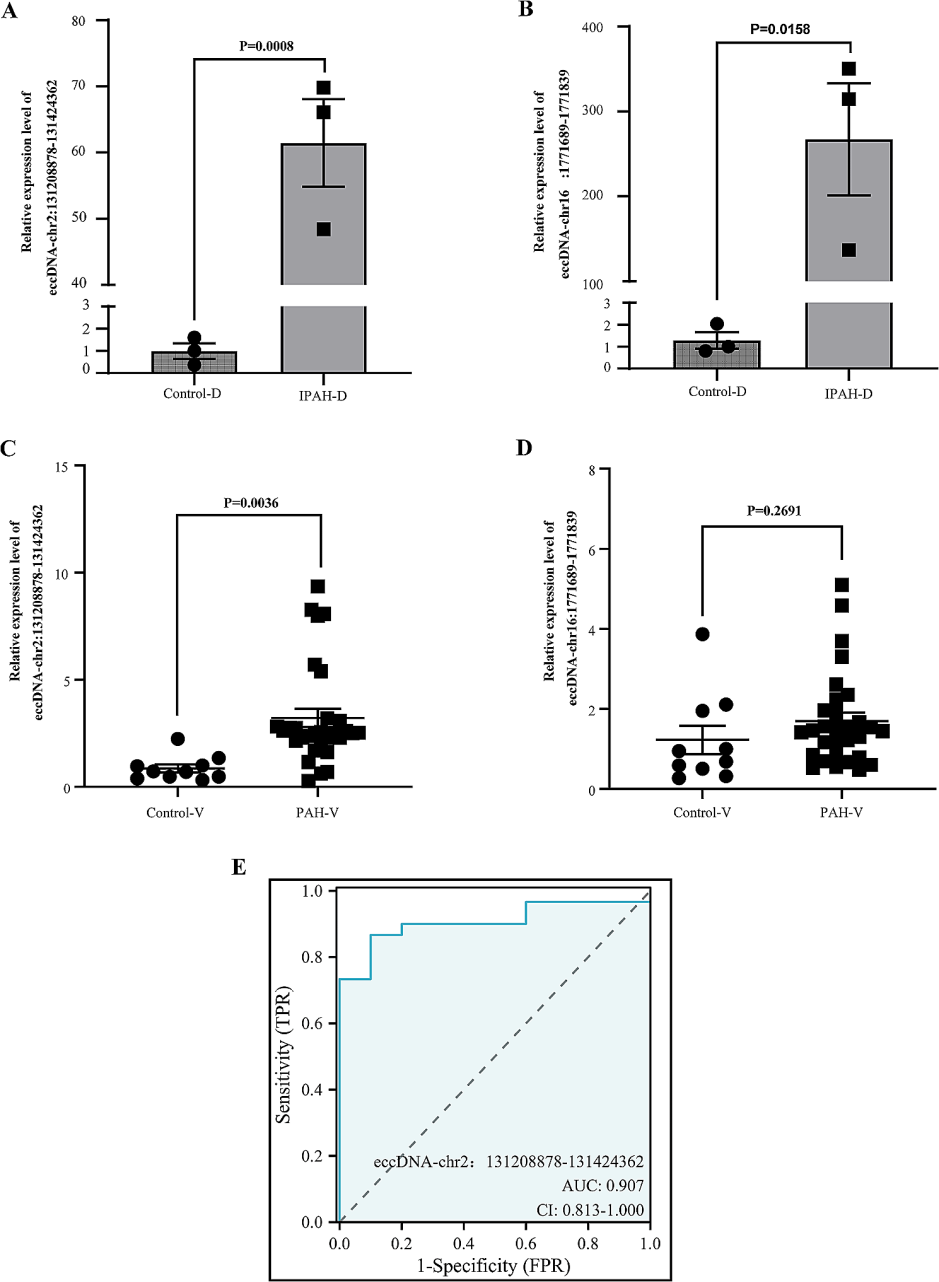
Expression levels of eccDNAs in patients with PAH and control participants. **A-B** Expression levels of two selected eccDNAs in patients with IPAH and control participants in the discovery group (patients, *n* = 3; controls, *n* = 3). **C-D** Expression of eccDNA-chr2:131208878–131,424,362 and eccDNA-chr16:1771689–1,771,839 in patients with PAH and control participants in the validation group (patients, *n* = 30; controls, *n* = 10). **E** The ability of serum eccDNA-chr2:131208878–131,424,362 level to diagnose PAH was assessed using receiver operating characteristic curve analysis

In the discovery cohort, two eccDNAs were chosen for further examination based on the gene-related functions anticipated by bioinformatics analysis and the level of eccDNA expression. These eccDNAs were named after the location of their genetic origin such as eccDNA-chr2:131208878–131,424,362 [27]. The qPCR results showed that the expression level of eccDNA-chr2:131208878–131,424,362 (*t* = 9.315, *P* = 0.0008) and eccDNA-chr161771689-1771839 (*t* = 4.027, *P* = 0.0158) in IPAH was significantly higher than that in the healthy controls, which was consistent with our Circle-Seq results (Fig. 4A-B). A further 30 patients with PAH and 10 healthy participants were recruited as a validation cohort, and we performed qPCR amplification on serum samples from this cohort to detect the expression level of eccDNA-chr2:131208878–131,424,362 and eccDNA-chr16:1771689–1,771,839 (see Supplementary Table S6 for quality control data). The expression level of serum eccDNA-chr2:131208878–131,424,362 (*t* = 14.932, *P* = 0.004) in patients with PAH was significantly higher than that in the healthy group (Fig. 4C). However, the verification of eccDNA-chr16:1771689–1,771,839 (*t* = 14.932, *P* = 0.269) was not ideal (Fig. 4D). When the sex differences in serum eccDNA levels were considered, the levels of serum eccDNA-chr2:131208878–131,424,362 were significantly elevated in patients with PAH compared with that in control subjects in each sex (Supplementary Fig. S3A–B). However, the levels of serum eccDNA-chr16:1771689–1,771,839 did not differ between the sexes (Supplementary Fig. S3C–D). Receiver operating characteristic curve analysis showed that serum eccDNA-chr2:131208878–131,424,362 had superior diagnostic ability to distinguish between healthy participants and patients with PAH. At the optimal expression cutoff value of 1.4848, the sensitivity and specificity of serum eccDNA-chr2:131208878–131,424,362 was 86.67% and 90%, respectively (Fig. 4E). These results demonstrated that serum eccDNA-chr2:131208878–131,424,362 could clearly distinguish patients with PAH from normal healthy controls, indicating the potential of this eccDNA as a plasma biomarker for PAH.

### Spearman’s correlation analysis of serum eccDNA-chr2:131208878–131,424,362 and clinical variables

**Fig. 5:**
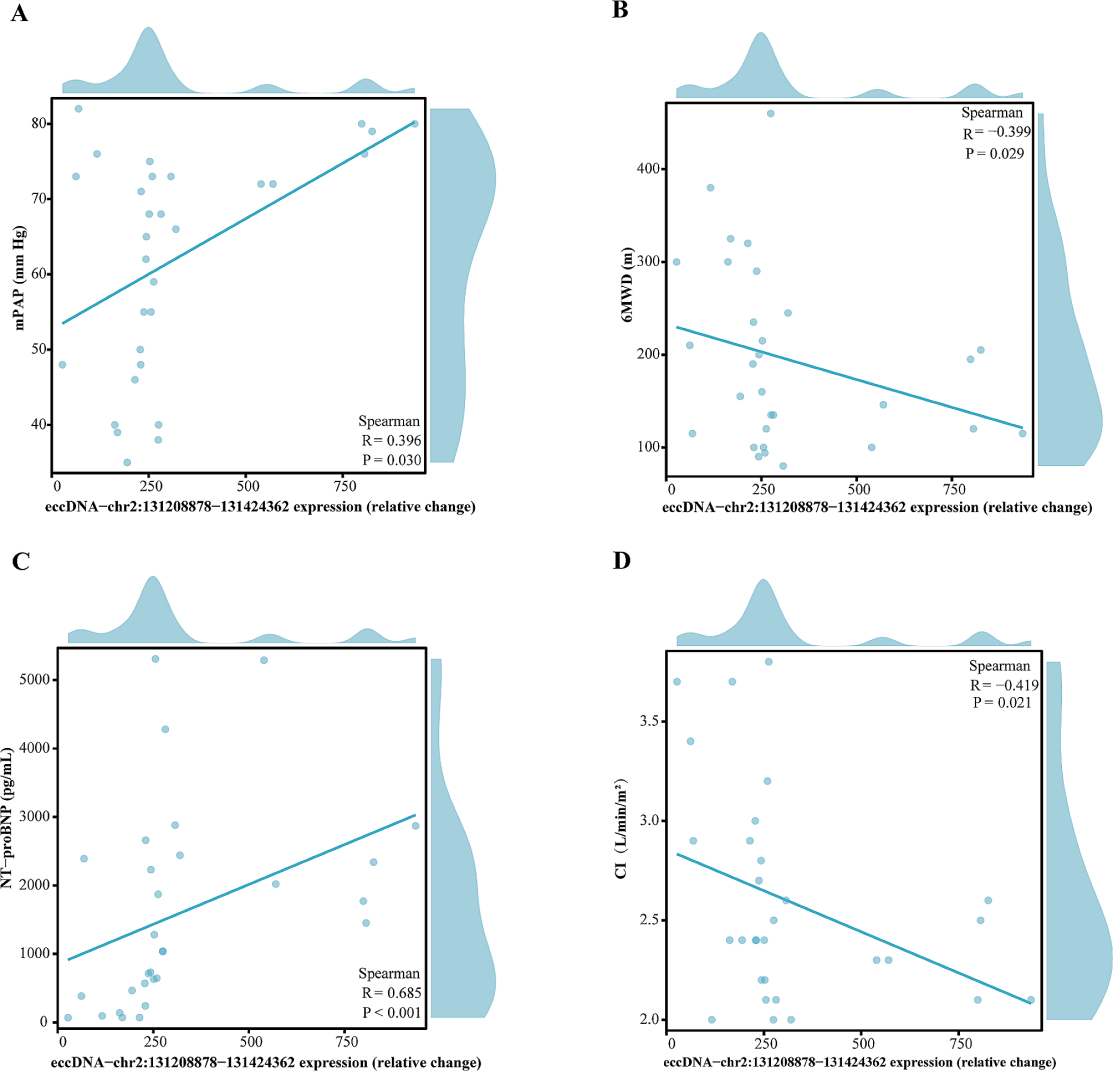
Correlation analysis between eccDNA-chr2:131208878–131,424,362 and selected baseline clinical characteristics in patients with PAH. Scatter plots of the relationship between serum eccDNA-chr2:131208878–131,424,362 expression and mean pulmonary artery pressure (mPAP) **(A)**, six minutes walking distance (6MWD) **(B)**, N-terminal pro-B-type natriuretic peptide (NT-proBNP) **(C)** and cardiac index (CI) **(D)**

As shown in Table 1, the clinical variables, including serum NT-proBNP, UA, high-density lipoprotein and right ventricular diameter were significantly different between PAH patients and healthy subjects (*P* < 0.05). Further correlation analysis disclosed a significant association between serum eccDNA-chr2:131208878–131,424,362 and mPAP (*r* = 0.396, *P* = 0.03) (Fig. 5A), 6MWD (*r* = -0.399, *P* = 0.029) (Fig. 5B), NT-proBNP (*r* = 0.685, *P* < 0.001) (Fig. 5C) and CI (*r* = − 0.419, *P* = 0.021) (Fig. 5D). However, there was no correlation between eccDNA-chr2:131208878–131,424,362 and other clinical characteristics (Table 2). When the correlation with sex was considered, we conducted further analysis for different genders. The results showed that serum eccDNA-chr2:131208878–131,424,362 was correlated with CI (*r* = -0.429, *P* = 0.046), NT-proBNP (*r* = 0.628, *P* = 0.002), Total bilirubin (*r* = 0.514, *P* = 0.015) and Cr (*r* = 0.557, *P* = 0.007) in the female group (Supplementary Table S7). In addition, serum eccDNA-chr2:131208878–131,424,362 was correlated with 6MWD (*r* = -0.333, *P* = 0.043), mPAP (*r* = 0.881, *P* = 0.007), and BUN (*r* = -0.786, *P* = 0.028) in the male group (Supplementary Table S8). The findings indicated that the eccDNA-chr2 131,208,878–131,424,362 in serum could serve as a new biomarker for diagnosing PAH in patients.

**Table 1:** Baseline characteristics of patients with PAH and healthy control subjects

Characteristics	Control subjects (*n* = 10)	PAH patients (*n* = 30)	P value
Age (years), mean ± sd	51.6 ± 6.1319	47.633 ± 15.865	0.262
Sex, n (%)			0.690
Female	6 (60%)	22 (73.33%)	
Male	4 (40%)	8 (26.67%)	
HR (beats/min), median (IQR)	70 (66, 78.5)	69 (65.25, 75.25)	0.790
BMI (kg/m^2^), median (IQR)	21.955 (21.332, 23.125)	22.02 (20.755, 24.703)	0.938
NYHA class, n (%)			N/A
I - II	N/A	7 (23.33%)	
III - IV	N/A	23 (76.67%)	
6MWD (m), median (IQR)		175 (116.25, 242.5)	N/A
**Hemodynamic**			
mPAP (mmHg), median (IQR)	N/A	67 (48.5, 73)	N/A
PCWP (mmHg), median (IQR)	N/A	9 (7, 11.75)	N/A
PVR (wood units), mean ± sd	N/A	9.6287 ± 5.605	N/A
CI (L/min/m^2^), median (IQR)	N/A	2.4 (2.2, 2.875)	N/A
**Biochemical markers**			
NT-proBNP (pg/mL), median (IQR)	38 (33.25, 55.25)	1160 (491, 2377.5)	**< 0.001**
Troponin I (ng/mL), median (IQR)	0.018 (0.01525, 0.0205)	0.0165 (0.007275, 0.0215)	0.435
D-dimer (ug/L), median (IQR)	347 (317.25, 461.75)	583 (262, 895.25)	0.246
Total bilirubin (µmol/L), median (IQR)	13.65 (10.8, 15.425)	11.85 (9.275, 17.275)	0.864
ALT (U/L), median (IQR)	18 (12.75, 22.5)	11 (9, 22)	0.324
AST (U/L), median (IQR)	25.5 (21.25, 26.75)	20 (16, 23)	0.054
Triglyceride (mmol/L), median (IQR)	1.485 (0.965, 1.7475)	1.155 (0.8625, 1.47)	0.281
High-density lipoprotein (mmol/L), mean ± sd	2.512 ± 1.0959	1.284 ± 0.46964	**0.006**
Low-density lipoprotein (mmol/L), mean ± sd	1.467 ± 0.52915	1.7763 ± 0.43843	0.074
BUN (mmol/L), median (IQR)	4.65 (3.525, 5.55)	4.95 (3.725, 5.775)	0.790
Cr (umol/L), median (IQR)	61 (50.5, 77)	59.5 (48.5, 74)	0.650
UA (umol/L), mean ± sd	214 ± 57.329	438.87 ± 137.41	**< 0.001**
**Echocardiography**			
LVEF (%), median (IQR)	77.5 (75.25, 79.5)	71 (65.25, 77.75)	0.063
Right atrial diameters (mm), mean ± sd	34.4 ± 4.4272	33.9 ± 3.0212	0.690
Right ventricular diameters (mm), median (IQR)	31.5 (30, 32)	36 (34.25, 38.75)	**< 0.001**
Left atrial diameters (mm), mean ± sd	31.6 ± 3.2387	29.967 ± 3.3578	0.187
Left-ventricular end-diastolic diameter (mm), mean ± sd	36.8 ± 2.044	35.733 ± 3.8141	0.406
Pulmonary artery diameter (mm), mean ± sd	28.2 ± 3.2592	29.345 ± 2.3188	0.234
Mitral orifice flow velocity (m/s), mean ± sd	0.83 ± 0.19465	0.77433 ± 0.23485	0.504
Aortic orifice velocity (m/s), median (IQR)	1.45 (1.1875, 1.575)	1.4 (1.2, 1.6)	0.962
Pulmonary valvular orifice velocity(m/s), mean ± sd	1.2 ± 0.27889	1.145 ± 0.21043	0.514

Definition of abbreviations: HR = heart rate; BMI = body mass index; NYHA = New York Heart Association; 6MWD = 6-minute-walk distance; mPAP = mean pulmonary arterial pressure; PCWP = pulmonary capillary wedge pressure; PVR = pulmonary vascular resistance; CI = cardiac index; NT-proBNP = N-terminal pro–brain natriuretic peptide; ALT = alanine aminotransferase; AST = aspartate aminotransferase; BUN = blood urea nitrogen; Cr = creatinine; UA = uric acid; LVEF = left ventricular ejection fraction; N/A = not applicable

**Table 2:** Correlations of eccDNA-chr2:131208878–131,424,362 with various parameters

Parameter	Correlation coefficient	*P*-Value
Age	0.095	0.619
BMI	0.193	0.307
6MWD	-0.399	**0.029**
mPAP	0.396	**0.03**
PCWP	0.320	0.084
PVR	0.281	0.132
CI	-0.419	**0.021**
NT-proBNP	0.685	*P* < 0.001
Troponin I	0.241	0.200
Total bilirubin	0.305	0.101
ALT	0.025	0.896
High-density lipoprotein	0.196	0.298
BUN	-0.163	0.389
Cr	0.352	0.057
UA	0.306	0.101
LVEF	-0.152	0.422
Right atrial diameters	-0.139	0.464
Right ventricular diameters	-0.091	0.633
Pulmonary artery diameter	0.290	0.127
Mitral orifice flow velocity	-0.159	0.402
Pulmonary valvular orifice velocity	-0.149	0.432

Definition of abbreviations: BMI = body mass index; 6MWD = 6-minute-walk distance; mPAP = mean pulmonary arterial pressure; PCWP = pulmonary capillary wedge pressure; PVR = pulmonary vascular resistance; CI = cardiac index; NT-proBNP = N-terminal pro–brain natriuretic peptide; BUN = blood urea nitrogen; Cr = creatinine; UA = uric acid; LVEF = left ventricular ejection fraction

## Discussion

Despite the utilization of different invasive and non-invasive techniques for diagnosing PAH, right heart catheterization continues to be considered the most reliable method [28]. However, the use of catheterization entails a higher chance of cardiopulmonary failure and may not always be feasible for continuous and repeated assessment [20]. In addition, although echocardiography is a useful non-invasive diagnostic technique for patients with PAH, this method exhibits a low rate of diagnosis and a high standard error of estimate [29]. Hence, there is an urgent need to develop a non-invasive technique for the diagnosis of PAH and the follow-up assessment of PAH.

Recent studies have indicated that epigenetic modifications may be associated with the pathogenesis of PAH and may serve as hopeful focus for diagnosing and treating the disease [30, 31]. Advances in sequencing technology have led to the discovery that eccDNAs are involved in a wide range of biological processes, including intercellular communication, intercellular genetic heterogeneity, regulation of immune response, and drug resistance generation, and as biomarkers for disease diagnosis and prognosis [10, 32–34]. One such method, Circle-Seq [35], is a purification technique that enables the identification and analysis of eccDNAs through high-throughput sequencing, and can aid in research on PAH [23, 25]. Using Circle-Seq, we identified a significant presence of eccDNAs in our PAH samples. These eccDNAs shared some features (e.g., length distribution, GC contents, and genomic distribution) with previously characterized eccDNAs [23, 25, 26, 36]. Despite the absence of notable distinctions in the characteristics mentioned, the size distribution of eccDNAs in all PAH samples showed a distinctive peak when compared to the healthy participants. In our study, the eccDNA distributions show a peak around 132 bp to 175 bp, which could be a distinctive characteristic of PAH when compared to eccDNAs found in healthy somatic tissues (with peaks at 100 bp and 5 kb) and plasma from pregnant women (with peaks at approximately 202 bp and 338 bp) [23, 37].

The functions of eccDNAs still need to be completely understood, particularly for the small eccDNAs that are under 1000 bp, which made up the majority of our sequencing findings [38]. In contrast to larger eccDNAs (> 100 kb), smaller eccDNAs (< 100 kb) are common in human cells [39, 40] and it has been proposed that eccDNAs circulating in the blood are ideal biomarkers because they are more stable than linear DNA [37]. In this study, based on Circle-Seq, we characterized the eccDNA profile of patients with PAH for the first time and identified a new eccDNA, eccDNA-chr2:131208878–131,424,362. In the discovery cohort, this eccDNA was significantly upregulated in the IPAH group compared to healthy controls. Therefore, we concluded that eccDNA-chr2:131208878–131,424,362 may have clinical value in PAH. Consequently, we utilized a larger number of clinical samples (the validation cohort) to validate the clinical potential of eccDNA-chr2:131208878–131,424,362. Elevated serum eccDNA-chr2:131208878–131,424,362 had high sensitivity (86.67%) and specificity (90%) for diagnosing PAH, suggesting that eccDNA-chr2:131208878–131,424,362 is a novel and non-invasive biomarker for diagnosing PAH. Furthermore, analysis of clinical variables revealed that eccDNA-chr2:131208878–131,424,362 expression correlated significantly with mPAP, 6MWD, NT-proBNP and CI, which had been reported to correlate with PAH progression [41, 42].

When analyzing the findings in this study, the following limitations should be considered: (1) We did not follow up the patients or serially assess the expression level of eccDNA-chr2:131208878 − 13,142,436, and PAH prognosis cannot be established. (2) The comprehensive mechanism of eccDNA-chr2:131208878–131,424,362 in PAH pathobiology has not yet been elucidated. However, recent studies have demonstrated the carcinogenic role of synthetic eccDNA containing microRNA-17-92 in the progression of liver cancer [43, 44]. The gastric cancer tissues that have elevated levels of eccMIRs were capable of generating operational miRNA molecules and enhancing cancer advancement through the stimulation of cell proliferation and aggressive characteristics [45]. In our study, GO and KEGG pathway analyses showed that differentially expressed eccDNA-associated mRNA genes in PAH were significantly enriched in calcium channel activity, the MAPK pathway, and the wnt signaling pathway, which have been extensively explored in the studies of PAH [46–49]. Therefore, we speculate that blocking the pathways for eccDNA generation may provide a novel strategy for the treatment of PAH with an aberrant high level of eccDNA production, and this will be the focus of our forthcoming investigations.

## Conclusion

We revealed for the first time the landscape and characteristics of eccDNAs in patients with PAH. Higher serum eccDNA-chr2:131208878–131,424,362 expression was associated with the incidence of PAH, suggesting that this eccDNA may be an emerging biomarker for PAH diagnosis. Further research to better understand the upregulation and functional characteristics of eccDNA-chr2:131208878–131,424,362 may generate novel therapeutic targets in PAH.

### Electronic supplementary material

Below is the link to the electronic supplementary material.

Supplementary Material 1

Supplementary Material 2

Supplementary Material 3

Supplementary Material 4

Supplementary Material 5

Supplementary Material 6

Supplementary Material 7

Supplementary Material 8

Supplementary Material 9

## Data Availability

The datasets analyzed during the present study can be obtained from the corresponding author upon a reasonable inquiry.

## Abbreviations

CO: cardiac output
CI: cardiac index
EccDNA: extrachromosomal circular DNA
GO: Gene Ontology
KEGG: Kyoto Encyclopedia of Genes and Genomes
MAPK: mitogen-activated protein kinase
mPAP: mean pulmonary arterial pressure
NYHA: New York Heart Association
NT-proBNP: N-terminal pro-B-type natriuretic peptide
PAH: pulmonary arterial hypertension
PVR: pulmonary vascular resistance
6MWD: six minutes walking distance
TnT: troponin T
UA: uric acid
