# Supplementary material for: Differential expression and analysis of extrachromosomal circular DNAs as serum biomarkers in pulmonary arterial hypertension
Source: Respir Res. 2024 Apr 25;25:181. doi: 10.1186/s12931-024-02808-z (PMC11046951; doi:10.1186/s12931-024-02808-z)
Supplement: Supplementary file 1 — Supplementary Material 1 [file 12931_2024_2808_MOESM1_ESM.docx]

**Supplementary Table 3 The quality control data of circle-sequencing.**

| **Sample** | **Raw reads** | **Clean reads** | **Clean Rate** | **Q30 (%)** |
| --- | --- | --- | --- | --- |
| Control 1 | 119,628,582.00 | 116,224,886.00 | 97.155% | 89.64% |
| Control 2 | 119,232,406.00 | 119,206,948.00 | 99.979% | 90.17% |
| Control 3 | 123,274,028.00 | 119,206,948.00 | 96.701% | 90.11% |
| IPAH 1 | 119,628,582.00 | 116,224,886.00 | 97.155% | 90.31% |
| IPAH 2 | 120,796,664.00 | 120,774,880.00 | 99.982% | 90.19% |
| IPAH 3 | 122,209,052.00 | 120,796,664.00 | 98.844% | 89.64% |
