# Supplementary material for: Differential expression and analysis of extrachromosomal circular DNAs as serum biomarkers in pulmonary arterial hypertension
Source: Respir Res. 2024 Apr 25;25:181. doi: 10.1186/s12931-024-02808-z (PMC11046951; doi:10.1186/s12931-024-02808-z)
Supplement: Supplementary file 2 — Supplementary Material 2 [file 12931_2024_2808_MOESM2_ESM.docx]

**Supplementary Table 6.**

**DNA QC Report.**

**DNA Quantification and Quality Assurance by NanoDrop ND-1000**

| **Sample ID** | **Sample Name** | **OD260/280 Ratio** | **OD260/230 Ratio** | **Conc.  (ng/μl)** | **Volume  (μl)** | **Quanity  (ug)** | **QC result** |
| --- | --- | --- | --- | --- | --- | --- | --- |
| 1 | G1 | 1.85 | 1.84 | 34.05 | 40 | 1.36 | pass |
| 2 | G2 | 1.84 | 1.89 | 31.56 | 40 | 1.26 | pass |
| 3 | G3 | 1.86 | 1.83 | 26.86 | 40 | 1.07 | pass |
| 4 | G4 | 1.83 | 1.84 | 21.01 | 40 | 0.84 | pass |
| 5 | G5 | 1.84 | 1.85 | 22.05 | 40 | 0.88 | pass |
| 6 | G6 | 1.88 | 1.84 | 32.4 | 40 | 1.30 | pass |
| 7 | G7 | 1.87 | 1.81 | 28.35 | 40 | 1.13 | pass |
| 8 | G8 | 1.80 | 1.86 | 29.13 | 40 | 1.17 | pass |
| 9 | G9 | 1.87 | 1.86 | 27.47 | 40 | 1.10 | pass |
| 10 | G10 | 1.84 | 1.89 | 30.21 | 40 | 1.21 | pass |
| 11 | G11 | 1.87 | 1.80 | 29.5 | 40 | 1.18 | pass |
| 12 | G12 | 1.83 | 1.85 | 24.44 | 40 | 0.98 | pass |
| 13 | G13 | 1.86 | 1.86 | 20.19 | 40 | 0.81 | pass |
| 14 | G14 | 1.87 | 1.82 | 24.73 | 40 | 0.99 | pass |
| 15 | G15 | 1.88 | 1.83 | 20.02 | 40 | 0.80 | pass |
| 16 | G16 | 1.89 | 1.83 | 31.78 | 40 | 1.27 | pass |
| 17 | G17 | 1.82 | 1.89 | 33.65 | 40 | 1.35 | pass |
| 18 | G18 | 1.83 | 1.85 | 28.59 | 40 | 1.14 | pass |
| 19 | G19 | 1.85 | 1.83 | 33.57 | 40 | 1.34 | pass |
| 20 | G20 | 1.82 | 1.81 | 30.89 | 40 | 1.24 | pass |
| 21 | G21 | 1.89 | 1.88 | 20.51 | 40 | 0.82 | pass |
| 22 | G22 | 1.81 | 1.83 | 23.06 | 40 | 0.92 | pass |
| 23 | G23 | 1.83 | 1.86 | 30.73 | 40 | 1.23 | pass |
| 24 | G24 | 1.85 | 1.88 | 20.22 | 40 | 0.81 | pass |
| 25 | G25 | 1.84 | 1.84 | 31.43 | 40 | 1.26 | pass |
| 26 | G26 | 1.86 | 1.83 | 21.31 | 40 | 0.85 | pass |
| 27 | G27 | 1.87 | 1.80 | 33.6 | 40 | 1.34 | pass |
| 28 | G28 | 1.81 | 1.87 | 26.64 | 40 | 1.07 | pass |
| 29 | G29 | 1.87 | 1.86 | 26.68 | 40 | 1.07 | pass |
| 30 | G30 | 1.85 | 1.85 | 21.6 | 40 | 0.86 | pass |
| 31 | H1 | 1.88 | 1.86 | 24.63 | 40 | 0.99 | pass |
| 32 | H2 | 1.85 | 1.88 | 20.87 | 40 | 0.83 | pass |
| 33 | H3 | 1.84 | 1.89 | 23.04 | 40 | 0.92 | pass |
| 34 | H4 | 1.81 | 1.89 | 27.04 | 40 | 1.08 | pass |
| 35 | H5 | 1.86 | 1.88 | 25.21 | 40 | 1.01 | pass |
| 36 | H6 | 1.85 | 1.85 | 20.5 | 40 | 0.82 | pass |
| 37 | H7 | 1.89 | 1.86 | 20.54 | 40 | 0.82 | pass |
| 38 | H8 | 1.87 | 1.88 | 21.51 | 40 | 0.86 | pass |
| 39 | H9 | 1.86 | 1.85 | 20.47 | 40 | 0.82 | pass |
| 40 | H10 | 1.87 | 1.84 | 28.64 | 40 | 1.15 | pass |
