# Supplementary material for: Differential expression and analysis of extrachromosomal circular DNAs as serum biomarkers in pulmonary arterial hypertension
Source: Respir Res. 2024 Apr 25;25:181. doi: 10.1186/s12931-024-02808-z (PMC11046951; doi:10.1186/s12931-024-02808-z)
Supplement: Supplementary file 3 — Supplementary Material 3 [file 12931_2024_2808_MOESM3_ESM.docx]

**Supplementary Table 2.**

**Primers in this study.**

| **Gene** | **Primer** | **Sequence (5 ’ to 3’)** |
| --- | --- | --- |
| pGEX-5X-2 | Forward | GGGCTGGCAAGCCACGTTTGGTG |
|  | Reverse | CCGGGAGCTGCATGTGTCAGAGG |
| chr2:131208878-131424362 | Forward | CATGCCTCGCGTCGTATAG |
|  | Reverse | CCAGACTCAGAATTCGTGCC |
| chr16:1771689-1771839 | Forward | AGTCTTGCTCTGTCG |
|  | Reverse | GTACTTTGGGAGGCCG |
