# Supplementary material for: Differential expression and analysis of extrachromosomal circular DNAs as serum biomarkers in pulmonary arterial hypertension
Source: Respir Res. 2024 Apr 25;25:181. doi: 10.1186/s12931-024-02808-z (PMC11046951; doi:10.1186/s12931-024-02808-z)
Supplement: Supplementary file 6 — Supplementary Material 6 [file 12931_2024_2808_MOESM6_ESM.docx]

**Supplementary Table 1.**

**Information for specimens**

Three patients with incidental IPAH and three healthy subjects were recruited for eccDNA-seq. Validation was performed with 30 patients with PAH and 10 healthy control subjects. The collected blood samples were immediately quick-frozen in liquid nitrogen.

**For eccDNA-seq**

|  | **Sample**  **No.** | **Age**  **(year)** | **Sex** | **NYHA class** | **mPAP (mmHg)** | **Sample**  **source** |
| --- | --- | --- | --- | --- | --- | --- |
| Control | 1 | 69 | Female | N/A | N/A | serum |
| Control | 2 | 43 | Male | N/A | N/A | serum |
| Control | 3 | 55 | Female | N/A | N/A | serum |
| IPAH | 1 | 42 | Male | II | 65 | serum |
| IPAH | 2 | 31 | Female | II | 70 | serum |
| IPAH | 3 | 51 | Female | IV | 47 | serum |

Definition of abbreviations: eccDNA = extrachromosomal circular DNA; IPAH = idiopathic pulmonary arterial hypertension；NYHA  = New York Heart Association; mPAP = mean pulmonary arterial pressure; N/A =  not applicable.

**For validation**

|  | **Sample**  **No.** | **Age**  **(year)** | **Sex** | **NYHA class** | **mPAP (mmHg)** | **Sample**  **source** |
| --- | --- | --- | --- | --- | --- | --- |
| Control | 1 | 49 | Female | N/A | N/A | serum |
| Control | 2 | 52 | Male | N/A | N/A | serum |
| Control | 3 | 60 | Female | N/A | N/A | serum |
| Control | 4 | 42 | Female | N/A | N/A | serum |
| Control | 5 | 48 | Male | N/A | N/A | serum |
| Control | 6 | 62 | Male | N/A | N/A | serum |
| Control | 7 | 47 | Female | N/A | N/A | serum |
| Control | 8 | 56 | Male | N/A | N/A | serum |
| Control | 9 | 51 | Female | N/A | N/A | serum |
| Control | 10 | 49 | Female | N/A | N/A | serum |
| PAH | 1 | 55 | Female | IV | 62 | serum |
| PAH | 2 | 46 | Male | IV | 73 | serum |
| PAH | 3 | 64 | Male | IV | 80 | serum |
| PAH | 4 | 58 | Female | II | 82 | serum |
| PAH | 5 | 46 | Female | II | 46 | serum |
| PAH | 6 | 52 | Male | III | 48 | serum |
| PAH | 7 | 44 | Male | II | 75 | serum |
| PAH | 8 | 13 | Female | IV | 68 | serum |
| PAH | 9 | 66 | Female | IV | 73 | serum |
| PAH | 10 | 68 | Male | IV | 50 | serum |
| PAH | 11 | 25 | Female | III | 73 | serum |
| PAH | 12 | 29 | Female | II | 40 | serum |
| PAH | 13 | 62 | Female | IV | 71 | serum |
| PAH | 14 | 26 | Female | II | 55 | serum |
| PAH | 15 | 26 | Female | IV | 68 | serum |
| PAH | 16 | 31 | Female | IV | 80 | serum |
| PAH | 17 | 68 | Female | II | 38 | serum |
| PAH | 18 | 58 | Male | IV | 72 | serum |
| PAH | 19 | 32 | Female | IV | 55 | serum |
| PAH | 20 | 35 | Female | III | 39 | serum |
| PAH | 21 | 48 | Male | IV | 65 | serum |
| PAH | 22 | 56 | Female | III | 76 | serum |
| PAH | 23 | 61 | Female | III | 48 | serum |
| PAH | 24 | 57 | Female | II | 35 | serum |
| PAH | 25 | 51 | Female | IV | 66 | serum |
| PAH | 26 | 32 | Female | IV | 79 | serum |
| PAH | 27 | 70 | Female | IV | 40 | serum |
| PAH | 28 | 31 | Female | IV | 59 | serum |
| PAH | 29 | 63 | Male | III | 76 | serum |
| PAH | 30 | 56 | Female | IV | 72 | serum |

Definition of abbreviations: PAH = pulmonary arterial hypertension；NYHA  = New York Heart Association; mPAP = mean pulmonary arterial pressure; N/A =  not applicable.
