# Supplementary material for: Differential expression and analysis of extrachromosomal circular DNAs as serum biomarkers in pulmonary arterial hypertension
Source: Respir Res. 2024 Apr 25;25:181. doi: 10.1186/s12931-024-02808-z (PMC11046951; doi:10.1186/s12931-024-02808-z)
Supplement: Supplementary file 7 — Supplementary Material 7 [file 12931_2024_2808_MOESM7_ESM.docx]

**Supplementary Table7 Correlations of eccDNA-chr2:131208878-131424362 with various parameters in the female group.**

| **Parameter** | **Correlation coefficient** | **p‐Value** |
| --- | --- | --- |
| Age | -0.034 | 0.881 |
| BMI | 0.030 | 0.895 |
| 6MWD | -0.300 | 0.176 |
| mPAP | 0.180 | 0.432 |
| PCWP | 0.398 | 0.067 |
| PVR | 0.259 | 0.245 |
| CI | -0.429 | **0.046** |
| NT-proBNP | 0.628 | **0.002** |
| Troponin I | 0.299 | 0.177 |
| Total bilirubin | 0.514 | **0.015** |
| ALT | 0.570 | 0.800 |
| High-density lipoprotein | 0.142 | 0.529 |
| BUN | 0.010 | 0.966 |
| Cr | 0.557 | **0.007** |
| UA | 0.299 | 0.176 |
| LVEF | -0.004 | 0.986 |
| Right atrial diameters | -0.167 | 0.456 |
| Right ventricular diameters | -0.272 | 0.220 |
| Pulmonary artery diameter | 0.356 | 0.104 |
| Mitral orifice flow velocity | -0.102 | 0.651 |
| Pulmonary valvular orifice velocity | -0.207 | 0.354 |

Definition of abbreviations: BMI = body mass index; 6MWD = 6-minute-walk distance; mPAP = mean pulmonary arterial pressure; PCWP = pulmonary capillary wedge pressure; PVR = pulmonary vascular resistance; CI = cardiac index; NT-proBNP = N-terminal pro–brain natriuretic peptide; BUN = blood urea nitrogen; Cr = creatinine; UA = uric acid; LVEF = left ventricular ejection fraction.
