# Supplementary material for: Differential expression and analysis of extrachromosomal circular DNAs as serum biomarkers in pulmonary arterial hypertension
Source: Respir Res. 2024 Apr 25;25:181. doi: 10.1186/s12931-024-02808-z (PMC11046951; doi:10.1186/s12931-024-02808-z)
Supplement: Supplementary file 8 — Supplementary Material 8 [file 12931_2024_2808_MOESM8_ESM.docx]

**Supplementary Table8 Correlations of eccDNA-chr2:131208878-131424362 with various parameters in the male group.**

| **Parameter** | **Correlation coefficient** | **p‐Value** |
| --- | --- | --- |
| Age | 0.119 | 0.793 |
| BMI | -0.071 | 0.882 |
| 6MWD | -0.333 | **0.043** |
| mPAP | 0.881 | **0.007** |
| PCWP | -0.470 | 0.240 |
| PVR | 0.048 | 0.935 |
| CI | -0.419 | 0.301 |
| NT-proBNP | 0.667 | 0.083 |
| Troponin I | 0.192 | 0.649 |
| Total bilirubin | 0.000 | 1.000 |
| ALT | 0.096 | 0.821 |
| High-density lipoprotein | 0.048 | 0.935 |
| BUN | -0.786 | **0.028** |
| Cr | -0.635 | 0.091 |
| UA | 0.119 | 0.793 |
| LVEF | 0.036 | 0.933 |
| Right atrial diameters | -0.096 | 0.820 |
| Right ventricular diameters | -0.024 | 0.955 |
| Pulmonary artery diameter | 0.221 | 0.599 |
| Mitral orifice flow velocity | 0.048 | 0.910 |
| Pulmonary valvular orifice velocity | 0.024 | 0.977 |

Definition of abbreviations: BMI = body mass index; 6MWD = 6-minute-walk distance; mPAP = mean pulmonary arterial pressure; PCWP = pulmonary capillary wedge pressure; PVR = pulmonary vascular resistance; CI = cardiac index; NT-proBNP = N-terminal pro–brain natriuretic peptide; BUN = blood urea nitrogen; Cr = creatinine; UA = uric acid; LVEF = left ventricular ejection fraction.
