# Supplementary material for: Differential expression and analysis of extrachromosomal circular DNAs as serum biomarkers in pulmonary arterial hypertension
Source: Respir Res. 2024 Apr 25;25:181. doi: 10.1186/s12931-024-02808-z (PMC11046951; doi:10.1186/s12931-024-02808-z)
Supplement: Supplementary file 9 — Supplementary Material 9 [file 12931_2024_2808_MOESM9_ESM.pdf]

Supplementary Fig. 1-3

For

Differential expression and analysis of extrachromosomal circular DNAs as serum biomarkers in pulmonary arterial hypertension

Chun Zhang[1†], Qiang Du[1†], Xiao Zhou[1], Tianyu Qu[1], Yingying Liu[1], Kai Ma[1], Ziling Shen[1], Qun Wang[1], Zaikui Zhang[2], Ruifeng Zhang[1]*

Correspondence: Ruifeng Zhang

Email: zrf1977313@163.com

A

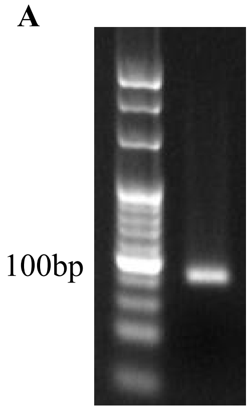

B

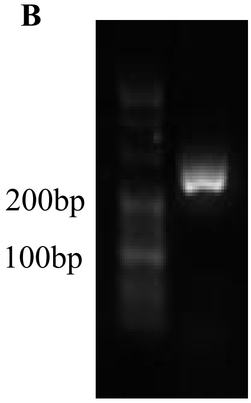

Supplementary Fig. 1 A. The amplified fragment size of chr16:1771689-1771839 was 90bp after separation by agarose gel electrophoresis. **B.** The amplified fragment size of chr2:131208878-131424362 was 235bp after separation by agarose gel electrophoresis.

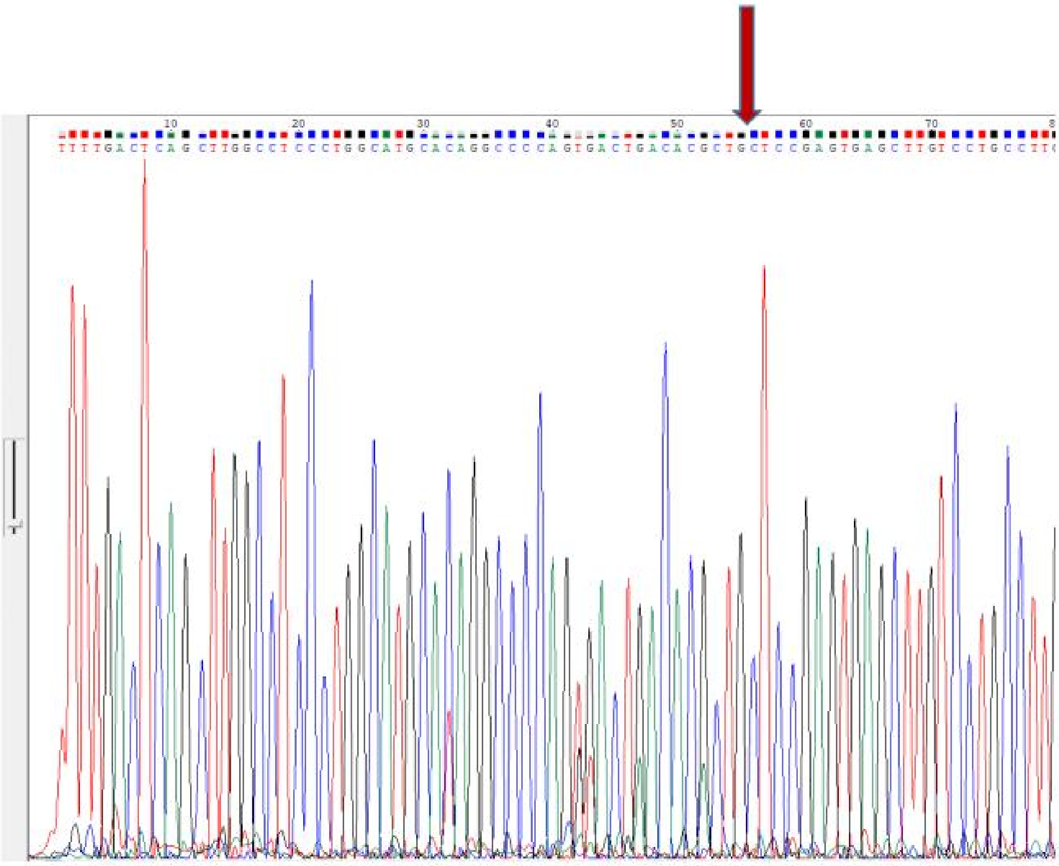

Forward primer: TTGACTCAGCTTGGCCTCC

Predicting sequence: >chr2:131208878-131424362

CTTGCTTGCCTGGAGGCAGACCACAGGCCGTCTTGAGGAAGACTTTATGTT

CAAGTACAGAAAGCAGCCAGGATTACCATCCAGGGGGGCCTTCTGTAGCC

CTGGCCAGACCTTGCAGAGGTGGCTGGTTGCTCTTTGACTCAGCTTGGCCT

CCCTGGCATGCACAGGCCCCAGGTACTAACATGTGCTCCAAGTGAGCTTGT

CCTGCATTGGCACGAATTCTGAGTCTGGCCAGGGTCACAGAAGGCCAAGT

CCCCTGGAAGGTTATCCTGGCTGCTTTCTGCACTTGAACATAAAGTCCTCCT

CAAGATGGCCTGTTGTCTGACTCTTGGCAACCAAGAAGCCTGCAGTGCCAT

ACGAGTTCTGAGGCATGGACTGGAGCCCC

Sequencing sequence: >R_235.1R.ab1

TTTTGACTCAGCTTGGCCTCCCTGGCATGCACAGGCCCCAGTGACTGACAC

GCTGCTCCGAGTGAGCTTGTCCTGCCTTGGCACAAATTCTATGTCTGGCCA

GGGCCACAGAAGGCCGAGTCCCCTGGATGGTAATCCTGGCTGCTTTCTGTA

CTTGAACGTAAAGTCCTCATCAAGACGGCCTGTGGTCTGCCTCTGCATCGA

AGTCAACCG

Breakpoint: TGTG

Supplementary Fig. 2 Sanger sequencing of PCR products confirmed the circular structure of eccDNA-chr2:131208878-131424362.

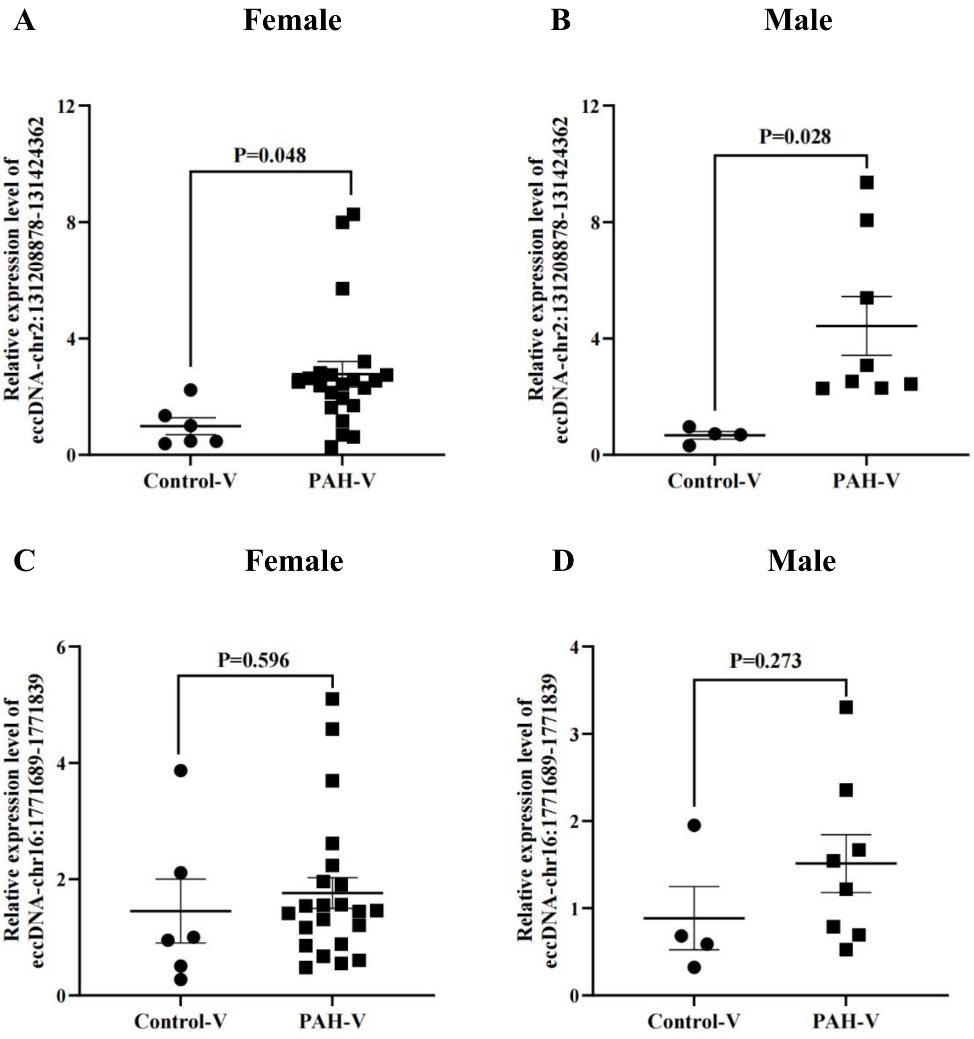

Supplementary Fig. 3 Expression levels of serum eccDNAs in patients with PAH and control participants in each sex. A-B Expression levels of eccDNA-chr2:131208878-131424362 in each sex. **C-D** Expression levels of eccDNA-chr16:1771689-1771839 in each sex.
